# The introduction, methods, results and discussion (IMRAD) structure: a Survey of its use in different authoring partnerships in a students' journal

**DOI:** 10.1186/1756-0500-4-250

**Published:** 2011-07-21

**Authors:** Loraine Oriokot, William Buwembo, Ian G Munabi, Stephen C Kijjambu

**Affiliations:** 1 Former Editor Makerere Medical Students Journal, Makerere University College of Health Sciences, New Mulago Hospital Complex, Kampala Uganda; 2 Department of Human Anatomy, School of Biomedical Sciences, Makerere University College of Health Sciences, New Mulago Hospital Complex, Kampala Uganda; 3 Dean's office, School of Medicine, Makerere University College of Health Sciences, New Mulago Hospital Complex, Kampala Uganda

**Keywords:** research, education, publications, undergraduate, IMRAD

## Abstract

**Background:**

Globally, the role of universities as providers of research education in addition to leading in main - stream research is gaining more importance with demand for evidence based practices. This paper describes the effect of various students and faculty authoring partnerships on the use of the IMRAD style of writing for a university student journal.

**Findings:**

This was an audit of the Makerere University Students' Journal publications over an 18-year period. Details of the authors' affiliation, year of publication, composition of the authoring teams and use of IMRAD formatting were noted. Data analysis gave results summarised as frequencies and, effect sizes from correlations and the non parametric test. There were 209 articles found with the earliest from 1990 to latest in 2007 of which 48.3% were authored by faculty only teams, 41.1% were authored by student only teams, 6.2% were authored by students and faculty teams, and 4.3% had no contribution from the above mentioned teams. There were significant correlations between the different teams and the years of the publication (*r_s _= *-0.*338 p *< 0.01 one tailed). Use of the IMRAD formatting was significantly affected by the composition of the teams (Χ^2 ^(2df) = 25.621, *p *< 0.01) especially when comparing the student only teams to the faculty only teams. (U = 3165 *r *= - 0.289). There was a significant trend towards student only teams over the years sampled. (*z *= -4.764, *r *= -0.34).

**Conclusions:**

In the surveyed publications, there was evidence of reduced faculty student authoring teams as evidenced by the trends towards students only authoring teams and reduced use of IMRAD formatting in articles published in the students' journal. Since the university is expected to lead in teaching of research, there is need for increased support for undergraduate research, as a starting point for research education.

## Background

Globally there is an increasing awareness of the importance of research for developing guidelines to direct social and economic interventions [1,2]. Research involves the critical analysis of each and every solution to a problem using the scientific method to identify the best evidence based solution for action at the time. Research is thus the foundation of evidence based practice [3,4]. Society expects universities to lead both the teaching and carrying out of research. This expectation has led to various policy recommendations and initiatives to promote research and innovation. An example of such a policy recommendation can be found in United States of America, where Gonzalez (2001) identifies the 1998 Boyer commission report encouraging universities to place more emphasis on undergraduate research experiences [5]. According to Laskowitz et al (2010), Stanford and Duke Universities have been running undergraduate research programmes for the last 40 years that instil in students an appreciation for rigorous research in academic medicine [6]. In Australia, students picked life skills like time management so long as they dealt with authentic science and had good supervision [7]. In Africa the demand for high quality research at undergraduate level of education, is yet to be met [8].

Research and innovation are critical for national social and economic development [2]. In response to the drive for more economic development, universities are redefining their roles and interactions with society by going from being the traditional storehouses of knowledge to becoming interactive knowledge hubs [9]. One way of ensuring that the Universities actually act as knowledge hubs is through promoting institutional visibility by encouraging research publication by students and faculty using internationally recognised scientific writing formats like Introduction, Methods, Results and Discussion, [IMRAD] [5,9,10]. In addition to visibility, the adoption of high quality international standards benefits the university by the creation of a pool of individuals who are conversant with scientific writing. Having such a pool of people supports Gonzales (2001) recognition that research takes place anywhere, and the "teaching of research is a role that is increasingly becoming the preserve of the university" [5]. This role of how research is taught is further extended with Gonzales (2001) arguing that undergraduate research is actually the beginning of a "five stage continuum of research education that ends with a post-doctoral experience" [5]. Research education promotes the uniform conduction, interpretation and response to research findings reported using familiar standard formats of scientific writing. Finally according to Aravamudhan and Frantsve (2009) research education and adoption of uniform formats of scientific writing promotes evidence based practice by improving information awareness, seeking and eventual application of new practices [3]. The rapid increase in the volume of very advanced knowledge and equally rapid changes in the working environment make it increasingly important to equip students with key research skills like scientific writing to keep abreast [3,4].

This paper looks at work done on the Makerere Medical Journal (MMJ), one of the students' journals at Makerere University. MMJ is run for and by the health professional student body at the former Faculty of Medicine (FoM) that with the School of Public Health became Makerere University College of Health Sciences (MakCHS) in 2008, [11-13] one of the Colleges of Makerere University (one of the oldest universities in Sub-Saharan Africa). With the University's Vision to become a leader in research in Africa, there is a high demand for research and scientific writing currently focusing on graduate research [14]. The effect of student faculty partnerships on undergraduate scientific writing to our knowledge is not well documented. The paper describes the role of student faculty partnerships in determining the formatting of the MMJ articles over an 18 year (1990-2007) period in the journal's existence.

## Methods

This was a retrospective audit of the Medical Journal MMJ, a publication of the health professional student body. The MMJ is a peer-reviewed publication that provides a platform for students to: share and exchange medical knowledge; develop writing and analytical abilities; promote awareness of students' contributions to health care; provide continuing medical education and foster valuable leadership and editorial skills. MMJ is published bi-annually and has been in existence from the early 1960's. The journal publishes: original articles, reviews, reports, letters to the editor, case reports, includes sections like: educational quizzes and cross word puzzles.

A hand search was made for complete journal volumes from various sources that included the Sir Albert Cook Library which is the main MakCHS library, personal collections and the journal editorial teams' files. For each article found, the following information was captured; the articles' authors and their affiliations, the use of the IMRAD format of writing papers, the composition of the authoring teams and the year of the publication. The data was analyzed using the Statistical Package for Social Sciences Inc. (version 12.0 for Windows, Chicago, Illinois) with the calculation of odd ratios and trend analysis being made with the aid of online Open Epi programme version 2.3.1 http://www.openepi.com [15]. The results were summarised as frequencies and presented in bar graphs and tables with calculation of odds ratios, effect sizes and trend analysis. Additional inferences were made with the aid of spearman's correlations and non parametric tests with the level of significance set as P value of less than 0.05.

Permission to use the data for this study was obtained from the editorial team for the journal. None of the authors' identification details were used during the analysis and the preparation of the paper.

## Results

Two hundred and nine (209) journal articles were found during the survey. The earliest publication was of the year 1990 and the most recent from 2007 from 13 volumes of the journal. Of the 209 articles 101/209 (48.3%) were authored by faculty only teams, 86/209 (41.1%) were authored by student only teams, 13/209 (6.2%) were authored by student faculty teams, and 9/209 (4.3%) had no affiliation indicated thus not classified into any of the above mentioned teams. Examination of the paper formatting revealed that only 70/209 (33.5%) of the papers were written using the IMRAD format. The number of articles found by year are summarised in Table 1, with the highest number of 33 in 2007 and lowest number of 5 seen in 1990. There was no significant change in the odds for IMRAD use over the years. (Mantel Hertz chi square for trend = 1.71 p value 0.1906). There were significant correlations between the different teams and the years of the publication r_s _= - 0.338 *(p *< 0.01 one tailed) and for teams and use of IMRAD formatting r_s _= - 0.265 *(p *< 0.01 one tailed).

**Table 1:** Use of IMRAD formatting in papers by the different types of authoring teams by year

Year	IMRAD Formatting	Team composition			Total
		**Student**	**Student and Faculty**	**Faculty**	

1990.00	Yes	1	0	1	2
	No	0	0	3	3
	**Total**	**1**	**0**	**4**	**5**
1991.00	Yes	1	1	10	12
	No	8	2	10	20
	**Total**	**9**	**3**	**20**	**32**
1992.00	Yes	1	3	7	11
	No	1	0	6	7
	**Total**	**2**	**3**	**13**	**18**
1993.00	Yes	0	0	1	1
	No	3	0	6	9
	**Total**	**3**	**0**	**7**	**10**
1994.00	Yes	0	1	11	12
	No	7	0	7	14
	**Total**	**7**	**1**	**18**	**26**
1995.00	Yes	0	0	3	3
	No	6	0	6	12
	**Total**	**6**	**0**	**9**	**15**
1998.00	Yes	0	0	3	3
	No	8	0	5	13
	**Total**	**8**	**0**	**8**	**16**
2005.00	Yes	2	3	3	8
	No	4	0	3	7
	**Total**	**6**	**3**	**6**	**15**
2006.00	Yes	7	1	0	8
	No	19	1	2	22
	**Total**	**26**	**2**	**2**	**30**
2007.00	Yes	3	1	6	10
	No	15	0	8	23
	**Total**	**18**	**1**	**14**	**33**

Use of the IMRAD formatting was significantly affected by the composition of the teams Χ^2 ^(2df) = 25.621, *p *< 0.001 using the Kruskal Wallis test. Post hoc Mann-Whitney team pair specific tests whose level of significance set at 0.025 showed that the use of IMRAD was not significant when comparing the mixed students-faculty with faculty only teams (U = 444, *r *= - 0.21), but, was significantly different when comparing the students only to faculty only teams (U = 3165, *r *= -0.289). Jonkheere's test revealed no trend in the use of IMRAD over the years sampled *J *= 10100, *z *= 0.211, *r *= 0.086. However there was a significant trend to more students only teams over the years sampled *J *= 6802, *z *= -4.764, *r *= -0.34.

## Discussion

The analysis of the data reveals that there is an increase in the number of students only teams submitting articles to the journal. This can be seen in the number of articles submitted which was highest at 33 in the 2007 journal. The increased interest in publication could be the result of a more aggressive editorial team or represent an increasing interest on the part of the student body in the value of research. Increase in undergraduate students interest in research is supported by the observation that globally there is increased interest in research at the undergraduate level as the beginning of research education [5]. The other factor that could support increased interest in research is the adoption of adult learning approaches to curriculum delivery by the FoM in 2003 [16].

Sadly the increased student interest in research is also accompanied by a significant trend towards reduced faculty engagement with students in research (*r *= - 0.34). Reduced faculty engagement also manifests in two other ways as seen in no change in the use of IMRAD over time (*J *= 10100, *z *= 0.211, *r *= 0.086) and the observation that the students only teams use IMRAD less than the faculty teams (U = 3165, *r *= -0.289). Even where the journal article had mixed student faculty teams there was no significant increase in the use of IMRAD when compared to faculty only teams (U = 444, *r *= - 0.21). Reduced engagement could also point to a different trend developing over time, there seems to be little support for undergraduate research in both the curricula and in extracurricular activities. This seems to have been going on for quite some time considering that most of the faculty were once students at this same university. Examining global trends as described by Gonzales (2001), research education has moved from being the premise of graduate students to a continuum that begins in undergraduate education [5]. Active support for undergraduate research is happening in more developed settings as is seen in the example of Duke and Stanford universities [6]. According to Lappato (2007) in undergraduate research experiences students' learn by being positively influenced by the process of investigation, and learning or from modelling higher order methods of thinking as they test and later communicate their research findings [17]. This makes the undergraduate research experiences a powerful tool for quickly increasing the number of high calibre researchers [18]. If one assumed that the use of the IMRAD format is a measure of scientific writing skill transfer then the deductions from the analysis of the data obtained from the student journal articles, suggests that for this population research is undergoing a slow but sure decline. This trend has been observed by other researchers concerning the African continent [8].

Given the powerful nature of the undergraduate research experiences as tools for grooming the next generation of scientists, it is important to look at other factors like the need for extra effort and time of faculty to transfer scholarly writing skills to students [19]. There is need for urgently exploration of mentoring undergraduates in research in line with global research education trends [5]. Some other interventions for consideration include using a training or mentoring programme each new MMJ editorial team [20], and use of the student assessment process as is done at the graduate level [8]. Using student assessment to promote scientific writing requires clear documentation of the different roles of the various participants and subsequent supervision, [21] in addition to the creation of an enabling environment using an institution wide research governance framework[22]. Given that individuals who participate in research as students will more likely continue to participate in research as faculty, it is important that all efforts are made to ensure that the students develop these vital scientific writing skills [19,23].

### Study limitations

This retrospective study of the MMJ had some limitations like: the poor journal publication record keeping, annual turnover of the volunteer student editorial board and use of abbreviated names made it difficult to identify some of the author details. Despite this, it was possible to obtain an adequate sample of the journal's publication for detailed analysis.

## Conclusions

This survey demonstrates that in the surveyed university population, faculty student partnerships are not producing the desired level of undergraduate research mentoring as evidenced by the reduced use of the IMRAD formatting in articles published in the MMJ. Given that the use of IMRAD is one of the core competencies for one to be an active member of the scientific community, inability to transfer this skill could help explain some of the identified gaps related to scientific writing in this university and Africa at large [8]. There is need to support undergraduate research in Africa using active mentoring programmes, providing training support for student journal editorial teams and use of innovative pro-scientific writing curricula. Such support could result in the quicker uptake and promotion of scientific writing and the reading of scientific literature in Africa over time.

## Competing interests

The authors declare that they have no competing interests.

## Authors' contributions

All the authors read and approved the final manuscript. LO: Participated in the conceptualisation, data collection and write up of the final paper. WB: Participated in all phases of the papers write up from conceptualisation, analysis to the final write up IGM: Participated in all phases of the study; conceptualization, data collection, analysis and write up. SCK: participated in the conceptualisation of the paper and review of the various drafts of the paper prior to submission.
